# Deep-sea turbulence evolution observed by multiple closely spaced instruments

**DOI:** 10.1038/s41598-021-83419-2

**Published:** 2021-02-16

**Authors:** Chu-Fang Yang, Wu-Cheng Chi, Hans van Haren

**Affiliations:** 1 Earth System Science Program, Taiwan International Graduate Program (TIGP), Academia Sinica and National Central University, Taipei, Taiwan; 2 Institute of Earth Sciences, Academia Sinica, Taipei, Taiwan; 3 College of Earth Sciences, National Central University, Taoyuan, Taiwan; 4 Royal Netherlands Institute for Sea Research (NIOZ), P.O. Box 59, 1790 AB Den Burg, The Netherlands

**Keywords:** Ocean sciences, Solid Earth sciences

## Abstract

Turbulent mixing in the deep ocean is not well understood. The breaking of internal waves on sloped seafloor topography can generate deep-sea turbulence. However, it is difficult to measure turbulence comprehensively due to its multi-scale processes, in addition to flow–flow and flow–topography interactions. Dense, high-resolution spatiotemporal coverage of observations may help shed light on turbulence evolution. Here, we present turbulence observations from four broadband ocean bottom seismometers (OBSs) and a 200-m vertical thermistor string (T-string) in a footprint of 1 × 1 km to characterize turbulence induced by internal waves at a depth of 3000 m on a Pacific continental slope. Correlating the OBS-calculated time derivative of kinetic energy and the T-string-calculated turbulent kinetic energy dissipation rate, we propose that the OBS-detected signals were induced by near-seafloor turbulence. Strong disturbances were detected during a typhoon period, suggesting large-scale inertial waves breaking with upslope transport speeds of 0.2–0.5 m s^−1^. Disturbances were mostly excited on the downslope side of the array where the internal waves from the Pacific Ocean broke initially and the turbulence oscillated between < 1 km small-scale ridges. Such small-scale topography caused varying turbulence-induced signals due to localized waves breaking. Arrayed OBSs can provide complementary observations to characterize deep-sea turbulence.

## Introduction

How turbulence generated by internal waves evolves and interacts in the deep-sea environment is still an unsolved question in oceanography. Turbulent motions are complex with a wide range of periods due to their multi-scale spatiotemporal interactions with each other and with the topography. This leads to difficulties in acquiring observations with sufficient spatiotemporal coverage to obtain a full view of turbulent motions in the ocean. Moored observatories on the seafloor are used for time-series measurements, e.g.,^1^. Dedicated single point 1D moored observations have contributed greatly to our understanding, e.g.,^2,3^, but with limited lateral spatial coverage. The combination of moored physical oceanographic measurements from temperature sensors and geophysical observations from ocean bottom seismometer (OBS) arrays may help to improve coverage for studying turbulence processes in the deep ocean.

In a deep-sea environment, internal waves are important sources of turbulent mixing generation, e.g.,^4^. Internal waves are 3D motions in density-stratified water and are ubiquitous in the ocean. Their periods can vary from hundreds of seconds to about one day^5^. The longest-period freely propagating internal waves are induced by inertial motions from adjustments of passing atmospheric disturbances and the Earth’s rotation^6^, while short-period internal waves are generated by buoyancy-gravity oscillations in the stable density stratification^7^. At frequencies in between, semidiurnal internal tides are the most common internal motions. When internal waves propagate toward sloped seafloor topography, the wave-topography interactions can induce turbulence and vertical mixing^2,8^. The spatial and temporal scales of turbulent motions range from hundreds of meters to millimeters and from hours to a fraction of a second (0.01 s), respectively^9–11^. The Brunt-Väisälä frequency, also called buoyancy oscillation frequency, which is a measure of the static stability in a stratified fluid environment to vertical displacements/oscillations with periods ranging from hours to minutes, generally determines the longest timescale of turbulent convection induced by vertical mixing^12^.

Common methods for measuring ocean turbulence include shipborne and free-fall profiling instruments, e.g.,^13,14^, in addition to moored acoustic devices, e.g.,^3,15^. Temperature measurements from moored sensors can be used as a tracer for density variations to study internal motions and to quantify the spatial scale and energy dissipation of turbulent mixing induced by internal waves breaking^8,16^. However, other instruments might also record relevant data.

OBSs not only detect earthquake signals but also non-earthquake signals related to natural and environmental processes, such as storm-induced microseisms, ocean infragravity waves, and long-period signals (> 10 s) related to ocean bottom flow^17,18^. Microseisms (0.08–0.5 Hz) are standing wind-induced ocean surface gravity waves^19^. Ocean infragravity waves are propagating surface gravity waves induced by the wind, but at lower frequencies, typically in the range 0.004–0.04 Hz^20,21^. Their wavelengths range from dozens to hundreds of kilometers^22^. Environmental signals induced by ocean bottom flow can be at longer periods, extending to hundreds of seconds^18^, which overlap the periods of large timescale turbulent mixing^23^. In the same frequency band, tilt motions related to ocean bottom flow have also been detected by many deployed OBSs^17,18^, but the source processes have not been systematically documented.

Geophysical observations related to internal-tide motions were detected by OBSs with three-component geophones^24^, which are commonly used to record high-frequency seismic motions. The strong translational signals with frequencies between 2 and 4 Hz, which repeatedly occurred nearly every semidiurnal tide cycle, coincided with the ambient temperature changes induced by the internal tides. Those temperature variations can be very different between stations a few dozens of kilometers apart, and have been speculated to be related to vertical mixing in stratified waters above.

Dense OBS-station arrays may help to understand the spatial evolution of turbulence in the deep sea through seismic array analysis techniques. Seismic array methods have been widely used to determine source direction and to estimate propagation speeds of plane waves arriving at the array, and to further image 3D structures between sources and the stations through which those waves pass. According to Rost and Thomas^25^, the basic methods most commonly used in seismology are cross correlation, beam-forming methods, seismic stacking, and frequency-wave number (FK) analysis. The dimensions and spacing of the array depend on the purpose of the study. Typically, 2D and 3D arrays are recommended for including different incidence angles of the plane waves^25^. The array spacing which determines the resolution of the imaged structures should be less than a quarter-wavelength of the observed plane waves.

**Figure 1:**
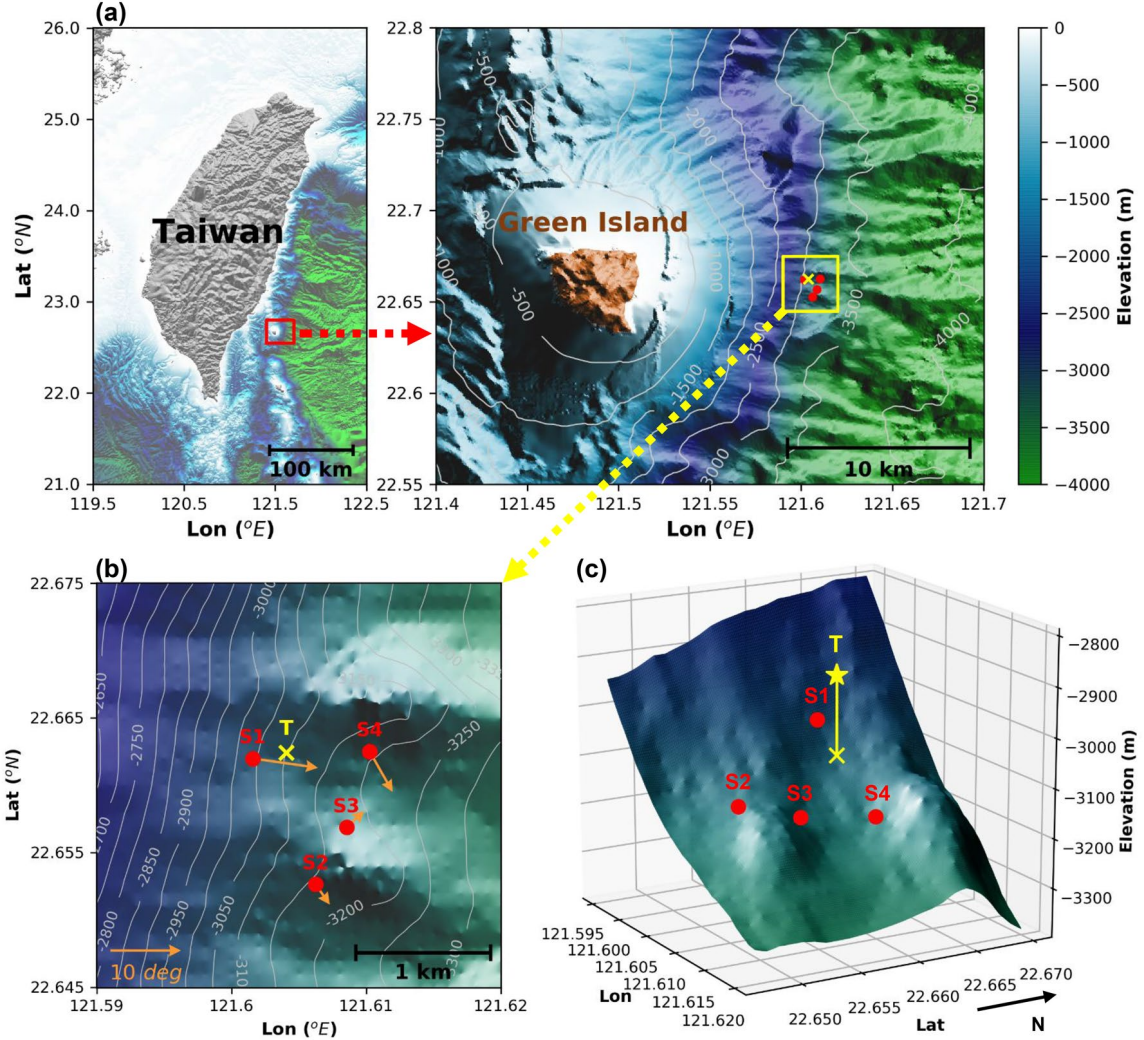
Location of experimental site. (**a**) A small-scale array within a 1 × 1 km footprint was deployed over continental slopes 10 km east of Green Island, Taiwan. (**b**) The array has four broadband ocean bottom seismometers (OBSs, red dots) and a 200-m long vertical thermistor string (T-string, yellow cross). Orange arrows show the magnitude and downslope direction of the local topography at each OBS. Gradients are computed from 100 × 100 m resolution interpolated bathymetry data from a multibeam survey. (**c**) 3D view of the deployed array showing the locations of the OBSs and the T-string with a Nortek AquaDopp acoustic current meter at 2936 m (star). Bathymetric maps were performed using Matplotlib graphic tool (version 2.1.2; URL: http://matplotlib.org) for Python.

Here, we use continuous waveforms from four broadband OBSs and high-resolution temperature data from a 200-m vertical thermistor string (T-string) mooring in a small array with a 1 × 1 km footprint to study deep-sea internal wave turbulence above the OBSs, instead of below them. The array was deployed over continental slopes offshore eastern Taiwan, 10 km east of Green Island (Fig. 1a), where internal waves are expected to break over the sloped topography facing the Pacific Ocean. Locally, the seafloor had slopes varying from 3 to 10°, calculated on 100-m scales. The OBSs were located between 3000 and 3200 m water depth with inter-station horizontal distances ranging from about 500 m to 1 km (Fig. 1b), and they are named from relatively shallow to deep water depth as S1, S2, S3, and S4. The multiple, spatially dense observations are able to track inertial internal motions and internal tides, whose horizontal wavelengths range from a few to several hundreds of kilometers^26^. The OBSs were deployed between September 2017 and April 2018. Inside the footprint of the array, 260 m east of S1 (Fig. 1c), a T-string mooring with a pressure sensor equipped Nortek AquaDopp acoustic current meter was deployed to measure water column temperature and pressure changes between June 2017 and April 2018.

The preliminary studies of this array presented by van Haren et al.^27^ document a typhoon-enhanced turbulence event using moored T-string data. A group of near-inertial motions recorded at the array site sustained for about 10 days when Category 4 tropical cyclone Typhoon Talim was passing northeast of the array. The interaction of the near-inertial waves with the sloping seafloor generated nonlinear internal waves which broke over the slopes and induced turbulent vertical overturns exceeding 200 m in height, leading to turbulent kinetic energy dissipation rates O(10^–7^) m^2^ s^−3^. Such overturning far exceeds turbulent overturns within Ekman depths O(10 m) induced by frictional shear mixing or by form drag at the seafloor.

In this paper, we use the same dataset as van Haren et al.^27^ and complement the study by focusing on the OBS observations. The broadband sensors can record a wide range of periods of the turbulent motion. In addition, the high spatiotemporal resolution of the OBSs and the vertical T-string mooring is expected to resolve the large-scale turbulent mixing induced by internal wave breaking. To demonstrate correlations between measurements from the two different types of instruments, we compare the time series of energy variations calculated from both datasets, and characterize them in different environmental conditions. We use cross-correlation of the energy variations from different OBS pairs to trace turbulence evolution in the array. We also apply seismic methods to calculate the back azimuth of the turbulent flow directions at each OBS, and to determine the impact of local topography on turbulent mixing. We discuss some turbulence processes that may produce the observed energy variations at the OBSs.

## Results

### Data overview

**Figure 2:**
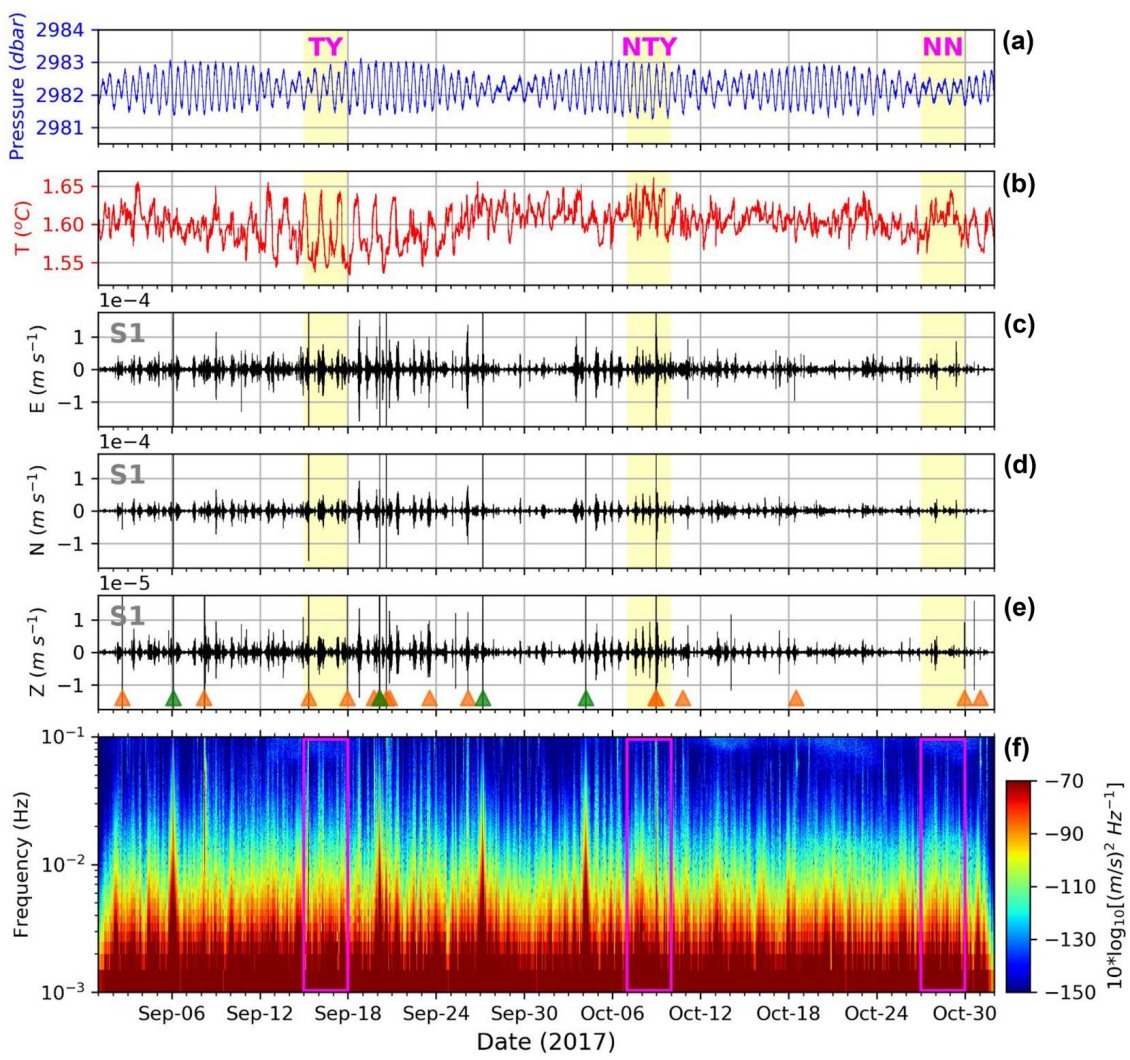
Two-month time series of several moored instrument parameters. (**a**) Pressure from current meter at 2936 m. (**b**) Temperature from T-string thermistor at 3131 m. (**c**) E–W component of velocity seismograms filtered between 0.00333 and 0.05 Hz observed at S1, the closest OBS to the T-string mooring. (**d**) Same as (**c**), but for the N–S component. (**e**) Same as (**c**), but for the vertical component. The vertical scale is one order of magnitude smaller than that in (**c**,**d**). Orange and green triangles indicate signals of earthquakes and gimbal system operations, respectively. (**f**) Spectrogram of vertical seismic signals at S1. Yellow shaded and magenta rectangular regions mark the time windows of the typhoon (TY), the non-typhoon (NTY), and the non-noisy (NN) periods that we analyzed in this study.

Continuous seismic recordings between 0.00333 and 0.05 Hz show many intermittent groups of tremor signals lasting for hours in September and October, 2017. These two months approximately at the end of summer may contain representative environmental signals related to tropical cyclone disturbances. Figure 2c–e depict velocity seismograms of the zonal (E–W), meridional (N–S), and vertical (Z) components at the shallowest OBS S1 as examples. The signals from the other OBSs are similar, at least to first order. Consistent with typical ground tilt signals, the amplitudes of the horizontal components are one to two orders of magnitude larger than those of the vertical components in the records of all OBSs. We can identify and exclude earthquake signals from the seismograms and spectrogram (Fig. 2f) by their relatively short duration (less than 100 s), high amplitude (at least two to three orders of magnitude larger than the other signals), and characteristic frequency range (from a fraction of a Hz to tens or hundreds of Hz). During the two-month-long records, the temperature variation recorded by the thermistor at 3131 m (Fig. 2b) was about 0.1 °C. The temperature variations from the thermistor (Fig. 2b) and the seismograms at S1 (Fig. 2c–e) have stronger oscillations in September than in October. Between September 12 and 24, the high seismic amplitudes and temperature variations correspond to Typhoon Talim approaching from the Northwest Pacific Ocean, its center passing northeast of the array. The temperature records show that typhoon-driven inertial waves passed through the array site^27^.

To characterize relationships between the environmental seismic signals and the internal wave-induced turbulence under different environmental conditions, we analyzed seismic and temperature data during a typhoon near-passage (TY) period, during a non-typhoon (NTY) period, and during a non-noisy (NN) period from September 15 to 17, October 7 to 9, and October 27 to 29, respectively. The TY and the NTY periods were selected based on their continuously high seismic-velocity fluctuations exceeding 10^–4^ m s^−1^ in the horizontal components. The NN period was selected for comparative analysis because it had relatively lower seismic-velocity fluctuations, with only two apparent short-duration groups of tremor signals. The NN period is also approximately during a neap surface tide (Fig. 2a). Three days of data from each period were selected for further detailed analysis due to computational limitations set by these high sampling rate datasets.

### Energy calculated from seismic and temperature data

**Figure 3:**
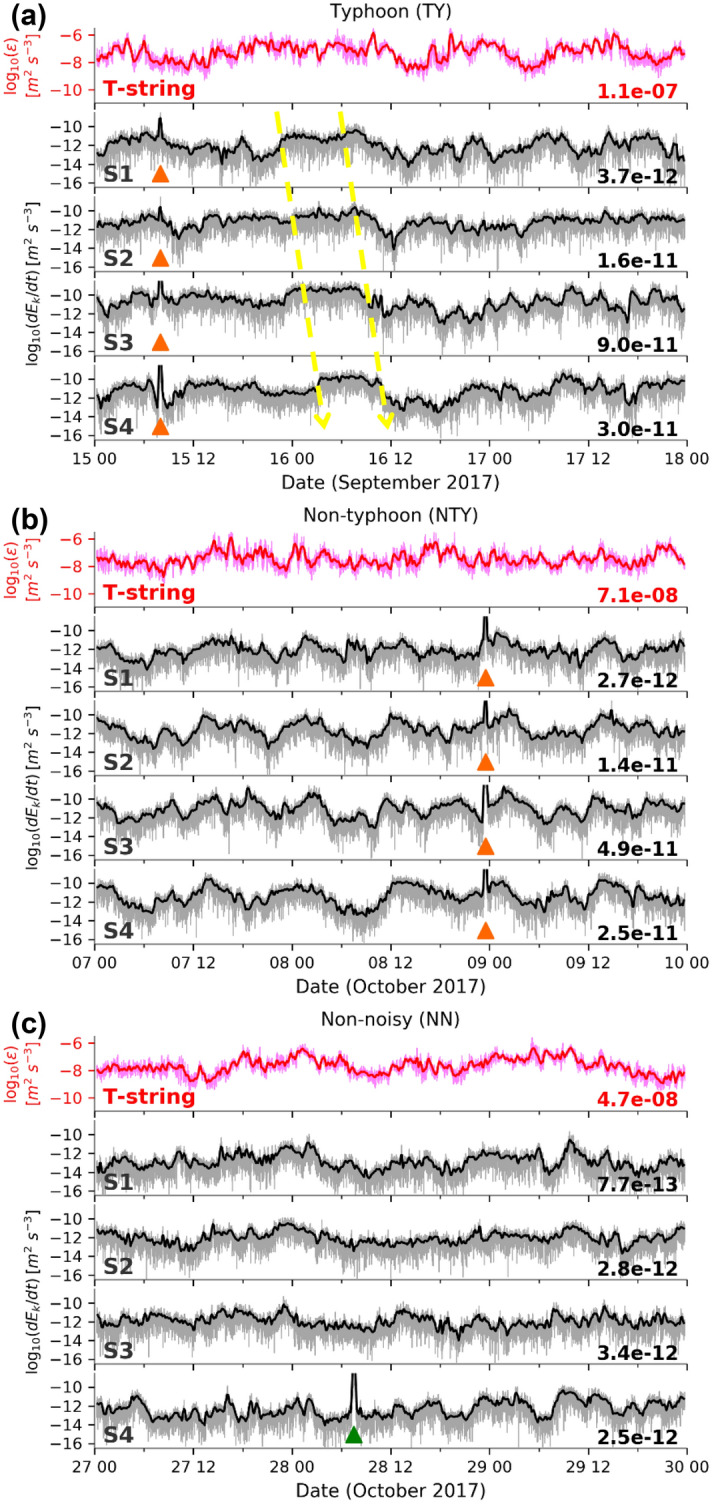
Three-day time series of vertically averaged over 200-m turbulent kinetic energy dissipation rate ($\varepsilon$) and the time derivative of kinetic energy (TDKE,$d{E}_{k}/dt$) calculated from T-string data and OBS velocity data, respectively, during: (**a**) Typhoon period (TY, September 15–17), (**b**) Non-typhoon period (NTY, October 7–9), and (**c**) Non-noisy period (NN, October 27–29) period. Dark and light lines in each panel show the raw and the 15-min time window (with half-window shifts) moving averaged time series, respectively. Orange and green triangles are the same as in Fig. 2e. The number at the bottom right of each panel is the time-averaged value of energy variations. Dashed yellow arrows show estimated horizontal apparent phase shifts.

Within this small array, the time series of the time derivative of kinetic energy (TDKE, see Sect. 4. Methods), which represents the apparent seismometer kinetic energy detection rate, at different OBSs are not identical, but have similar phase variations with a few hour shifts. To obtain the energy variations relevant to the timescales of turbulent motions close to buoyancy periods, the time series of energy variations was smoothed with a 15-min moving average with a half-window shift. The three day moving averaged TDKE variations at S1 generally show similar variations with time as the vertically averaged turbulent kinetic energy dissipation rate ($\varepsilon$, henceforth ‘dissipation rate’ in short, see Sect. 4. Methods) from the T-string in each period (Fig. 3). Higher TDKE correlates with higher dissipation rates, although several phase shifts and short-term discrepancies exist. During the TY period (Fig. 3a), the S1-TDKE and the dissipation rate show sustained high amplitudes between September 15, 12 UTC and September 16, 12 UTC, and higher energy was also detected at the deeper S3 and S4. The 15-min averaged TDKE variations have significant phase shifts at different depths of OBSs between September 15, 18 UTC and September 16, 12 UTC. The phases shifted sequentially from shallow S1 to deeper S4 during the first 12 h, and then similarly started phasing out during the following 6 h. The apparent horizontal phase speed estimated by the phase shifts from the TDKE of the OBSs is 0.04 m s^−1^. Compared with the time series of TDKE at S1 during the TY period (averaged value 3.7×10^–12^ m^2^ s^−3^), lower and more short-term energy variations were observed during NTY (Fig. 3b, averaged value 2.7 × 10^–12^ m^2^ s^−3^) and NN (Fig. 3c, averaged value 7.7×10^–13^ m^2^ s^−3^). During the NTY period, the TDKE variations contain several short-period fluctuations of less than 6 h duration at S1 while apparent semidiurnal variations were more dominant at S4. The time series of TDKE present different characteristics in short-term variations even among the OBSs located less than 1 km apart.

**Figure 4:**
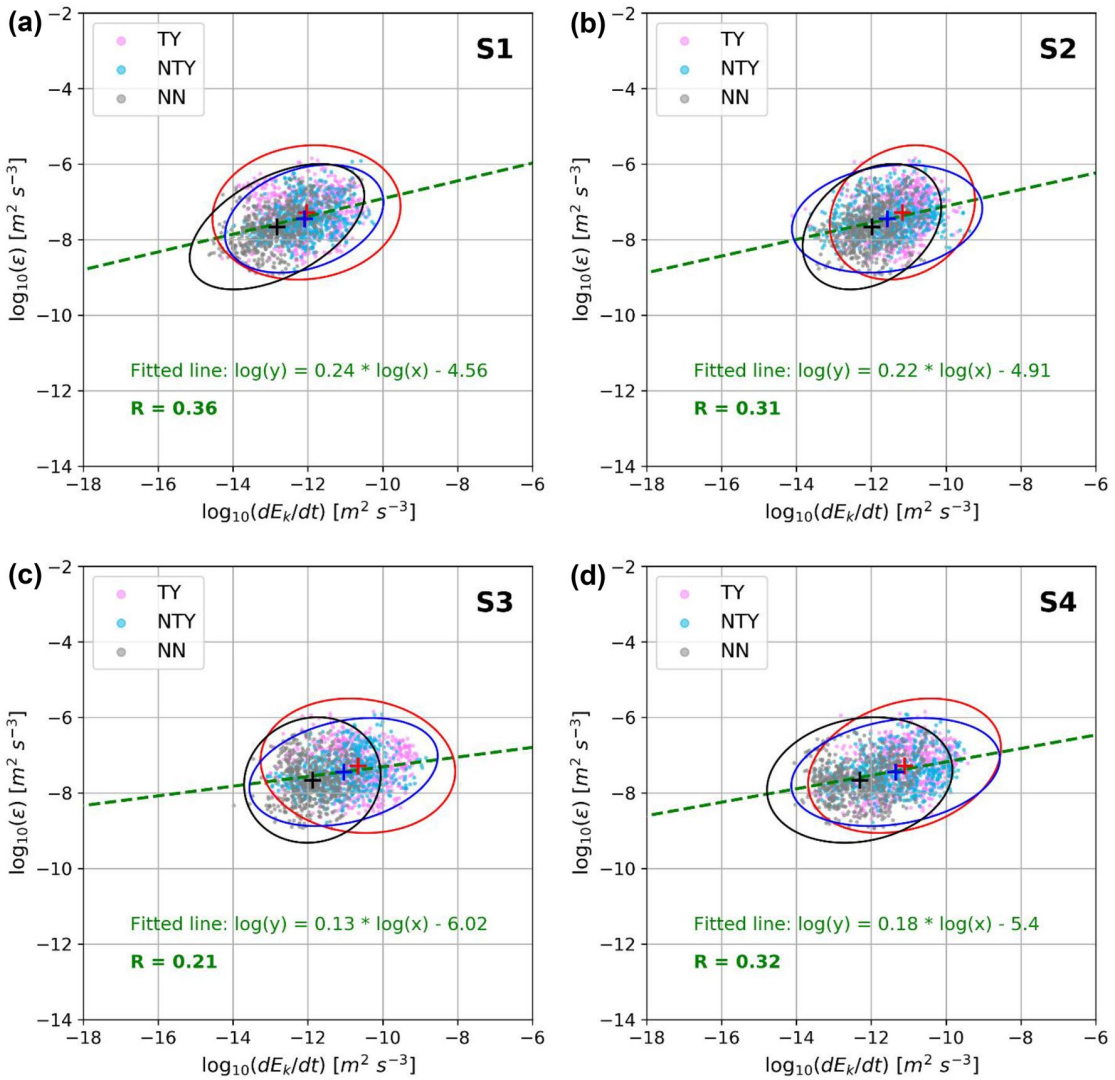
TDKE from the OBS (**a**) S1, (**b**) S2, (**c**) S3, and (**d**) S4 against T-string-inferred turbulent kinetic energy dissipation rate vertically averaged over 200-m during the TY (pink dots), the NTY (light blue dots), and the NN (gray dots) period. All datasets are from the three-day time series shown in Fig. 3, using a 15-min time window moving average with the half-window shift. Red, blue, and black crosses/ellipses show averaged values/three-sigma confidence intervals of the energy distributions, respectively. The dashed green line is the log–log regression line to fit all three-period energy distributions. R is the Pearson correlation coefficient between two datasets.

The TDKE at the different OBSs is in good agreement with the dissipation rates inferred from the T-string (Fig. 4). The TDKE (10^–14^–10^–9^ m^2^ s^−3^) is about three to five orders of magnitude smaller than the dissipation rate (10^–9^–10^–6^ m^2^ s^−3^). The dissipation rate is associated with higher TDKE during the TY period and with relatively lower TDKE during the NN period. The TDKE at S1 (Fig. 4a) has a higher correlation coefficient (R = 0.36) with the T-string dissipation rate. S4 (Fig. 4d) and S2 (Fig. 4b) have lower correlation coefficients (R = 0.32 and R = 0.31 respectively) than S1, while S3 (Fig. 4c), located furthest from the T-string, has the weakest correlations with T-string data (R = 0.21). These relationships suggest that local effects are dominant on turbulent disturbances in the array.

### Turbulence motion in the small array

Our objective was to detail the phase shifts of the energy variations associated with turbulence evolution during the TY period, and to determine whether the turbulence developing and dissipating over the slopes can be characterized by the OBS observations. The cross-correlation of the 15-min averaged time series of TDKE was performed on 3-h moving time windows with 15-min time shifts, in order to include timescales of the largest energy-containing turbulence scales but smaller than the buoyancy periods. The local buoyancy frequency ranges from 1 × 10^–4^ to 1 × 10^–3^ Hz at the array site. Turbulence is a spatially small-scale intermittent process, unlike both acoustic and elastic wave propagation. Thus, concurring high correlation coefficients of similar phases of energy variations between different pairs of OBSs likely indicate turbulent motions generated by the same system of waves breaking. Using the 3-h window cross correlations, longer-period turbulent motions may be difficult to track. For example, the downslope motions seen in the time series of energy variations between 18 UTC on September 15 and 12 UTC on September 16 (Fig. 3a) have apparent phase shifts of about 6 h between S1 and S4.

**Figure 5:**
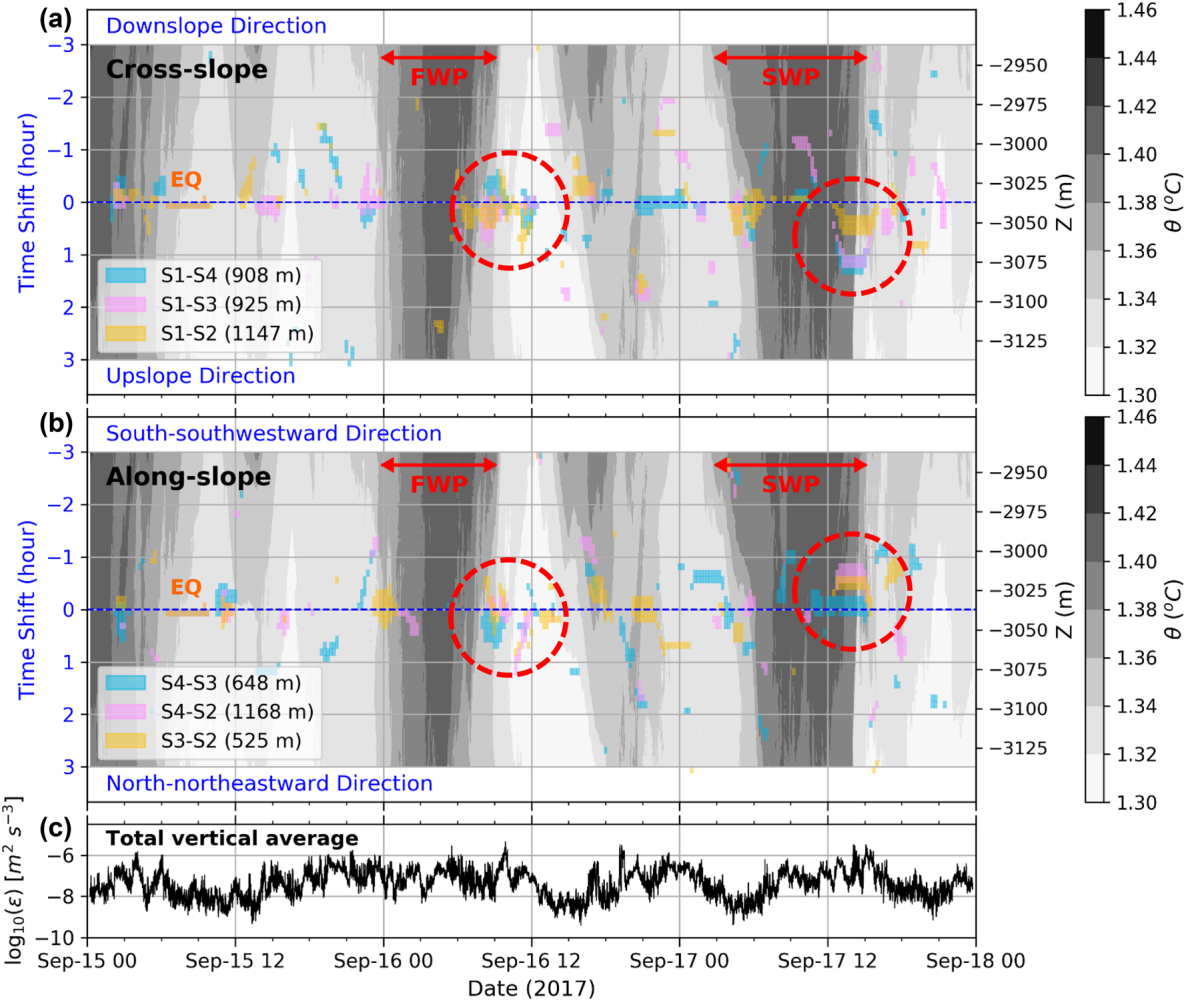
Cross-correlation of TDKE between different OBS pairs during the TY period over 3-h time windows moved with 15-min time shifts, for correlation coefficients greater than 0.6 (color shading; also indicated is the distance between the different pairs): (**a**) tracking cross-slope turbulent motions from S1 and (**b**) tracking along-slope turbulent motions in the north-northeast-south-southwest direction on the deep/downslope side of the array. The potential temperature $\theta$ depth time-series from the T-string (gray shading) shows two warm phases on September 16, from 00 to 09 UTC (first warm phase, FWP) and on September 17, from 03 to 15 UTC (second warm phase, SWP). (**c**) Total vertically averaged dissipation rate time-series inferred from T-string.

Turbulent motions in the cross-slope direction were tracked relative to S1 (Fig. 5a). Similarly, TDKE cross-correlations between S2, S3, and S4 can track turbulent flow along the north-northeast to south-southwest direction, roughly along the large-scale slope (Fig. 5b). The coincidence of high correlation coefficients with a consistent direction of movement between different OBS pairs suggests that a large-scale turbulent mixing event passed through the array, while measurements with inconsistent directions of movement might imply that multiple local-scale turbulent mixing events were induced simultaneously in the array.

During two inertial phases of the TY period, relatively warm water was transported to greater depths, almost reaching the seafloor. These phases took place on September 16, between 00 and 09 UTC (first warm phase, FWP), and on September 17, between 03 and 15 UTC (second warm phase, SWP). High correlation coefficients (> 0.6) between pairs of OBSs concurred over several hours during the transition from warm to cold phases and mostly coincided with high dissipation rates (> 10^–6^ m^2^ s^−3^, Fig. 5c). For example, high correlations were concomitant between six pairs of OBSs around the end of the FWP (September 16, 06 to 12 UTC) and the end of the SWP (September 17, 12 to 18 UTC). As the end of the FWP passed over the array and the temperature decreased, turbulent motions were induced on September 16, between 06 and 12 UTC (dashed red circle on September 16 in Fig. 5a,b). There were two disturbances around 09 and 12 UTC. The primary disturbances with a duration of about 3 h around 09 UTC show coincident positive and negative time shifts, implying multiple localized turbulent mixing events within the array. The following disturbances around 12 UTC which have positive time shifts of cross- and along-slope motions are northward upslope turbulence evolutions. On the other hand, the SWP resembles a frontal bore across the slope. When potential temperature dropped significantly by 0.4 °C between 12 and 15 UTC on September 17, energy was transported south-southwestward in the upslope direction (dashed red circle on September 17 in Fig. 5a,b). The apparent turbulent transport speeds estimated from the time shifts/time lags between S4–S3, S4–S2, and S3–S2 are about 0.2–0.5 m s^−1^ at the end of the SWP (Fig. 5b). This range of propagated speed is generally consistent with the phase speed of baroclinic inertial waves^6^. According to the apparent phase speeds and the energy high-correlation duration of 2 to 3 h, the apparent horizontal scales of the sustained turbulent motions associated with the inertial motions are estimated to be about 1.5 to 5.5 km.

Two weak internal motions with double inertial frequency (2f) were observed by the vertical T-string around 12 UTC on September 15 and 18 UTC on September 16. The disturbances induced by the 2f motions were shorter-duration localized turbulent mixing, compared with those at the end of the FWP and SWP.

### Local effects

Back azimuth tracking of OBS waveforms may help to characterize the turbulence source direction. Back azimuth tracking is a widely used seismic method to determine source direction by rotating two horizontal component waveforms to find the receiver-to-source back azimuth which gives the maximum sum of root-mean-square values. Following this method, the maximum amplitude of the turbulence-induced signal should be parallel to the average direction of turbulent flow, which was determined by rotating the horizontal velocity waveforms using a 15-min time window with a half-window shift over the three-day OBS records.

**Figure 6:**
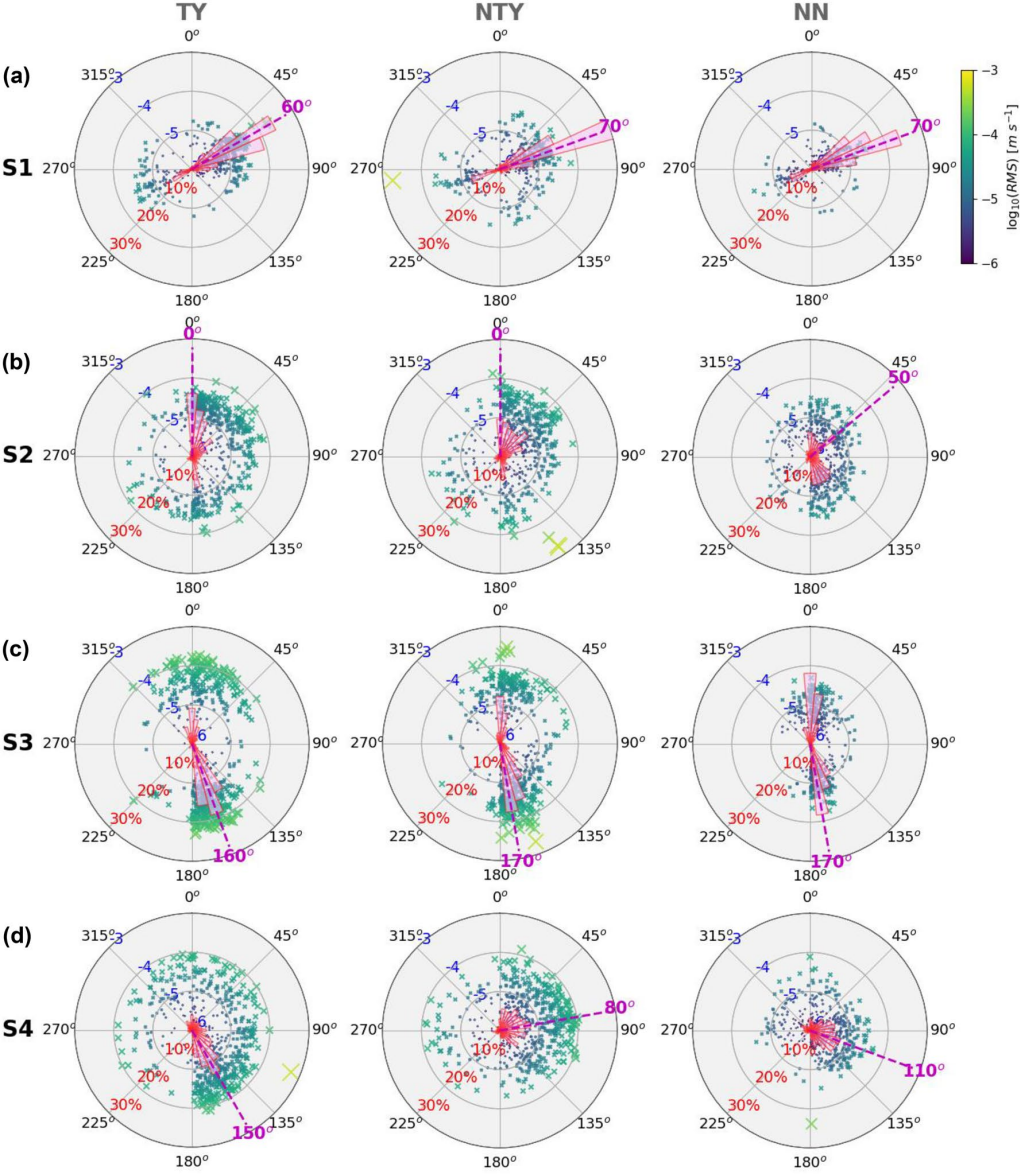
Back azimuths of turbulence sources determined from the maximum root-mean-square (RMS) of the rotated horizontal velocity waveform in every 15-min time window with half window shifts at OBS (**a**) S1, (**b**) S2, (**c**) S3, and (**d**) S4 during the TY, the NTY, and the NN periods. The colors of the crosses show the amplitude of the RMS. Pink bars show the statistical percentages of the back azimuths in every ten-degree sector at each OBS. The purple dashed line shows the maximum percentage of the back azimuths.

The majority of the back azimuths of the turbulence-induced signals at each OBS came from a similar direction during the three periods. For S1, the back azimuths were consistently oriented in an east-northeast (60–70°) direction (Fig. 6a), approximately along the downslope direction. The back azimuths of the signals detected by S2 (Fig. 6b) and S3 (Fig. 6c) were mostly oriented to the north-northeast (0–50°) and south-southeast (160–170°), respectively, which roughly point in the upslope directions of the two OBS locations. Although the back azimuths of the signals at S4 were more varied during the three periods, those signals mostly came from the local downslope direction of the station (Fig. 6d), like S1.

Varied azimuths determined from the 15-min time-window waveforms imply that interactions between the turbulent flow and seafloor topography are complicated over short periods of time and short distances. The unfocused azimuths at different OBSs make it difficult to determine a single breaker zone during each period. A nearly consistent azimuth at a particular OBS during different periods suggests that the turbulent motions were likely affected by local topography and depth. The overall statistical results show that most turbulent disturbances originated from downslope (east) of the array. This implies that the turbulent mixing was generated not only by internal waves breaking over the downslope area, but also by oscillatory motions of turbulence sloshing back and forth between the small-scale ridges on the east side of the array.

## Discussion

The OBS-measured signals with frequencies between 0.00333 and 0.05 Hz are related to turbulent motions induced by internal waves breaking in the deep sea. Although this frequency band is similar to the frequency range of ocean infragravity waves, the signals we study here have different characteristics from infragravity waves. The observed signals show significant temporal inconsistency at OBSs less than 1 km apart, while infragravity waves, typically having wavelengths of 10–100 km, are expected to show more consistency across the small OBS footprint of about 1 km horizontal distance.

It may be difficult to use seismically-detected environmental signals to quantify the kinetic energy of turbulent motion. Although the phase variations are similar, the underestimation of TDKE, compared to the T-string-inferred dissipation rates, may be caused by the narrower seismic frequency bandwidth we used. As a result, the TDKE from the filtered signals is insufficient to represent wider spectra of turbulent motions that include periods from 20 to 0.01 s. The TDKE thus presents only a fraction of the kinetic energy relative to the energy dissipated through turbulent mixing. However, turbulent motions are broadband and their spectra vary mostly linearly in the frequency domain, and usually do not illustrate frequency peaks. The TDKE, even though limited by bandwidth, illustrated similar phases with the turbulent kinetic energy dissipation rates derived from the T-string. Thus, a scaling factor may be applied to quantify turbulent kinetic energy dissipation rates from OBS data with a specific frequency band. However, this needs to be further investigated with more datasets as local site effects are likely important.

**Figure 7:**
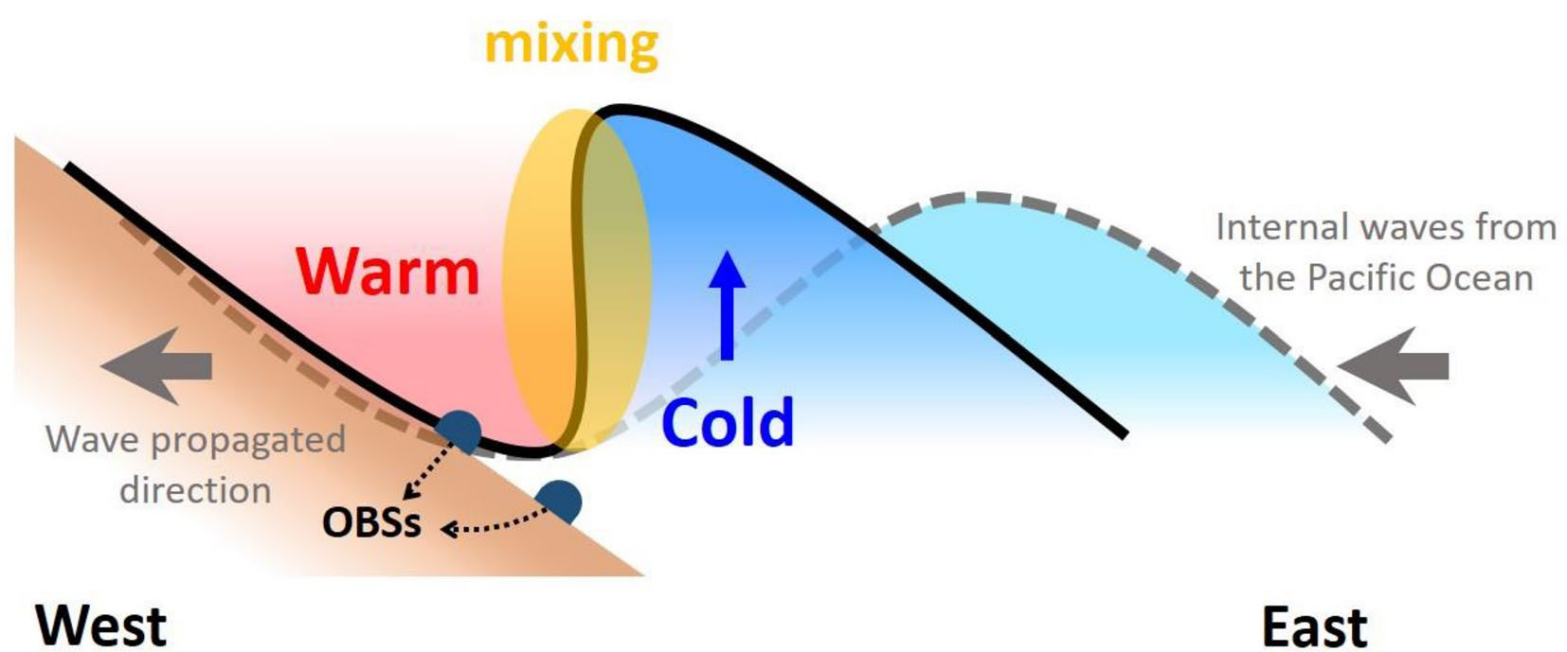
Illustration of turbulent mixing induced by a sloshing internal wave over a large slope. The dashed gray line shows the internal waveform propagating in the ocean interior. Less-dense warm water overlies denser cold water. The solid black line shows the deformation of the internal wave due to topography effects. The wave-breaking generates turbulent overturning and mixing (yellow shades) mainly in the transition from the warm to cold phase. Such motions are detectable at the seafloor by OBSs.

Large-scale turbulent motions within the OBS array are demonstrated by high correlation coefficients between different instrument pairs during the transition from warm to cold phases induced by internal waves. This observation suggests that turbulent mixing occurs through wave breaking processes^28–30^ in the phase transition of temperature changes due to internal waves sloshing over slopes (Fig. 7). As an internal wave propagates, the wave accompanies lower-density warm water above and relatively higher-density cold water below the interface. When a wave hits a slope, compressing the waveform from linear to non-linear and causing stacking and mixing of the interface between warm and cold water, the interaction leads to cold water being lifted by the slope, inducing wave overturning and breaking. Thus, turbulent mixing is more active in the transition from the warm to the cold phase, and may be enhanced and become a large-scale overturning event if a frontal bore system moves upslope. This is consistent with higher amplitude TDKE related to inertial motions observed during the TY period^27^.

Local topographic effects on turbulent motion can explain the scattered source back azimuths at different locations of the OBSs. Among them, S2 and S3, located on the flanks of a small ridge, had opposite directions of back azimuths. Both point toward the upslope direction of the small ridge. This may be caused by hydraulically controlled flow over local small ridges. Froude numbers ($Fr$) estimated by the T-string data are larger than the threshold for supercritical flow ($Fr$ > 1). They have maximum values of 6.3, 2.0, and 1.7 for the TY, NTY, and NN periods, respectively. The hydraulic jump of upward supercritical flow over slopes may generate strong turbulence near the top of the ridge^31^. Thus, both OBSs detect more disturbances over the respective ridge they are deployed on. Such processes might have been recorded in deep sea marine sediments, and can perturb biogeochemical signals within a short distance.

Turbulent flow consists of nonlinear 3D motions with a wide range of temporal scales (hours to 0.01 s). Internal waves interacting with the large and small ridges may result in more complicated turbulent motion. These complex multi-scale interactions lead to difficulties in predicting the occurrence of turbulence. This may explain the highly variable OBS-detected turbulence signals within the small array, and why some of the turbulent events could not be identified by cross-correlations. The untracked localized turbulent disturbances imply that the array we designed is not small enough to trace less than 100-m scale horizontal turbulent motions. A setup with more OBSs that are more densely distributed may help to provide higher-resolution observations at smaller scales. A smaller array with 10 to 100 m spacing, depending on the spatial scale of the turbulence to be characterized, is suggested for future studies of local submesoscale turbulence.

Turbulent motions near the deep seafloor may also influence teleseismic studies using presumed ground motions caused by faraway earthquakes. The frequency band of turbulence-induced environmental signals overlaps that of teleseismic surface wave signals (0.01–0.1 Hz), and thus can result in difficulties in determining OBS orientation using Rayleigh-wave polarization^32^ and for teleseismic tomography studies. In addition, the temperature perturbations on the seafloor induced by the turbulence might propagate to shallow sub-bottom depths, distorting the temperature profiles in the sediment measured by a thermal probe for marine heat flow measurements. Thus, dense OBS arrays in the future might provide complementary ways to study deep-sea turbulence processes, which can affect many different marine geophysical measurements. Field surveys combining arrayed OBSs and other geophysical and physical oceanographic instruments at one site may be needed to quantify such processes in the future.

## Methods

### Instrument settings

Three-component seismic data were recorded by OBSs built by the Institute of Earth Sciences of Academia Sinica in Taiwan^33,34^. Each OBS has a Nanometrics Trillium Compact broadband sensor mounted on a gimbal system, and velocity motions from the broadband sensor were recorded by an in-house digitizer with a 100 Hz sampling rate. The gimbal system regulates the horizontal level of the sensor and keeps the sensor’s vertical component aligned with the direction of gravity. The factory-designed flat response for the Trillium Compact broadband sensor is between 0.00833 to 50 Hz. Amplitudes of signals at frequencies above and below the flat response range are calibrated in the frequency domain using the instrument response function. Broadband seismometers detect both translational and rotational motions. The translational signals are dominant at frequencies in the flat response range while the sensors are more sensitive to rotational (or tilt) motions at frequencies below 0.00833 Hz^35^. Seismically detected tilt signals typically show much higher amplitudes on the horizontal component than the vertical component.

Temperature data were collected from sensors built at NIOZ, the Royal Netherlands Institute for Sea Research. These sensors provide high-resolution (precision < 0.0005 °C; noise level < 0.0001 °C) temperature data at a 0.5 Hz sampling rate^36,37^. The T-string had a Nortek AquaDopp acoustic current meter with pressure sensor at 2936 m and 101 T-sensors at 2-m intervals between 2937 and 3137 m. The deepest sensor was at 7 m above the seafloor.

### Data processing

We use turbulent kinetic energy dissipation rate and time derivative of kinetic energy calculated from the T-string and the OBSs, respectively, to analyze turbulence in the deep sea. To quantify the turbulence generated by internal waves breaking, temperature data are used as a tracer to calculate density variations and the stability of vertical displacements. The temperature-density relationship was obtained from a shipborne Conductivity Temperature Depth (CTD) profile measured near the T-string mooring site^27^. The turbulent kinetic energy dissipation rate $\varepsilon$, a measure of the energy dissipated through turbulent mixing, is calculated from depth displacements ($d$) referenced by a stable profile from a reordered temperature profile at every time step and buoyancy frequency ($N$) from the vertical density variations derived from temperature data^16^,

$$\varepsilon =0.64{d}^{2}{N}^{3}$$

The constant of 0.64 is obtained from (L_O_/d_rms_)^2^ = 0.8^2^, an empirical mean coefficient value^27,38^, where L_O_ is the Ozmidov length scale and d_rms_ is the root-mean-square displacement. For this study, to obtain turbulence-related signals from the OBSs, we band-pass filtered seismic waveforms between 0.00333 to 0.05 Hz. This frequency band includes both translational and rotational signals, and excludes signals from wind- and typhoon-induced microseisms due to standing waves and ultra-low frequency noise on the vertical component generated by temperature fluctuations^39^. Generation of low-frequency environmental signals are considered here as a result of turbulence disturbing the seismic sensors in the water. To get the same physical parameter to compare two datasets from instruments designed with different physical purposes, we calculated kinetic energy from the band-pass filtered seismic recordings by merging three (E–W, N–S, and vertical) components of kinetic energy from the velocity waveforms.

$${E}_{k}=\frac{1}{2}\left({{v}_{x}}^{2}+{{v}_{y}}^{2}+{{v}_{z}}^{2}\right)$$

We took the time derivative of the kinetic energy $\left( {{\text{TDKE}},\;{{dE_{k} } \mathord{\left/ {\vphantom {{dE_{k} } {dt}}} \right. \kern-\nulldelimiterspace} {dt}}} \right) .$ to obtain the time series of apparent seismometer kinetic energy detection rate for comparison with the dissipation rate calculated from the T-string data. The TDKE computed from the band-pass filtered seismic data contains only a portion of the kinetic energy of the turbulent motions because turbulence has a broader continuous bandwidth. However, in the frequency spectrum, the energy of homogeneous and isotropic turbulent motions is distributed as a function of wavenumber/frequency of eddies, following Kolmogorov’s Law of the turbulent cascade, allowing such band-limited observations to be used for comparison.
